# Phenolic compounds and antioxidant activity in *Cucurbita ficifolia* fruits, an underrated fruit

**DOI:** 10.3389/fnut.2022.1029826

**Published:** 2023-01-11

**Authors:** G. Moreno-Quiroga, J.E. Alba-Jiménez, E. N. Aquino-Bolaños, J. L. Chávez-Servia

**Affiliations:** ^1^Centro de Investigación y Desarrollo en Alimentos de la Universidad Veracruzana, Xalapa, Mexico; ^2^CONACyT-Centro de Investigación y Desarrollo en Alimentos, Universidad Veracruzana, Xalapa, Mexico; ^3^CIIDIR-Oaxaca, Instituto Politécnico Nacional, Santa Cruz Xoxocotlán, Mexico

**Keywords:** functional foods, antioxidant compounds, landraces, Mesoamerica, bioactive compounds

## Abstract

The fruits and seeds of *Cucurbita ficifolia* Bouché are sources of minerals, vitamins, and functional compounds with nutraceutical and preventive potential against cardiovascular diseases and diseases derived from eating disorders. *C. ficifolia* is native from Mesoamerica and is currently cultivated in temperate zones from Mexico to South America and Asia. This study evaluated the fruit mesocarps of *C. ficifolia* for physicochemical parameters, antioxidant activity, and phenolic compound contents in a collection of farmers’ landraces. Germplasm is cultivated by traditional farmers in the temperate zones of two municipalities from Oaxaca, Mexico. The results show that the content of soluble solid contents (SSC), pH, total sugars (TS), and flavonoids are influenced by the fruit geographical origin (municipalities) and implicitly by their agroecological cultivation conditions (Huamelúlpam: SSC = 6.22 °Brix, pH = 5.44, TS = 0.52 mg G g^–1^, flavonoids = 1.24 mg CE g^–1^; Yanhuitlán: SSC = 6.69, pH = 5.33, TS = 0.55, flavonoids = 1.30). Among populations preserved by traditional farmers, significant differences, and wide variability were found for all parameters evaluated (Huamelúlpam: SSC = 4.9–7.3, pH = 5.5–5.8, TS = 0.4–0.7, protein = 5.8–11.4, polyphenols = 1.9–4.8, flavonoids = 1.0–1.5, DPPH = 4.3–10.6, and FRAP = 4.8–11.8; Yanhuitlán: SSC = 4.3–8.9, pH = 4.8–5.6, TS = 0.4–0.7, protein = 5.0–15.3, polyphenols = 1.9–4.9, flavonoids = 0.8–1.9, DPPH = 5.3–10.5, and FRAP = 4.5–12.6). Eight compounds were identified by UPLC-MS: L-phenylalanine, an amino acid that is regularly associated with proteins; vanillin, a phenolic aldehyde with its functional groups including aldehyde, hydroxyl, and ether; and six phenolic acids: 4-hydroxybenzoic acid, 4-hydroxyphenylacetic acid, vanillic acid, 4-coumaric acid, ferulic acid, and salicylic acid, all with potential health effects. The *C. ficifolia* fruit mesocarp has bioactive compounds with high antioxidant activity with the potential to both improve diet and to obtain other benefits against nontransmissible diseases derived from food and its associated risk factors.

## 1 Introduction

Less known horticultural plants have recently gained more popularity. They include high contents of nonnutritive, nutritive, and bioactive compounds such as flavonoids, phenolics, anthocyanins, phenolic acids, as well as, sugars, essential oils, carotenoids, vitamins, and minerals. Certain lesser-known fruits also have distinct flavors and tastes, excellent medicinal value, and health care functions (1–3).

**FIGURE 1:**
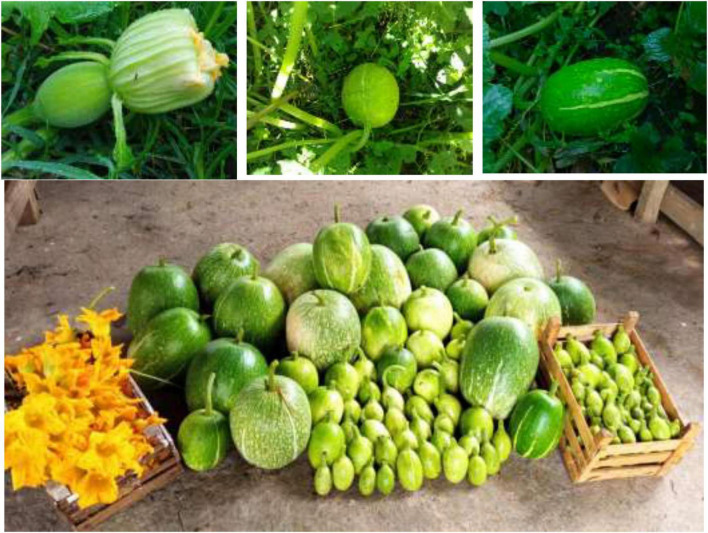
Fruits of *Cucurbita ficifolia* at different growth stages.

The specie *Cucurbita ficifolia* Bouché belongs to the *Cucurbitaceae* family and is distributed from southern Mexico to Central and South America in temperate to humid temperate climates at altitudes of 1,000–3,000 m. Both the immature and ripe fruits are widely consumed by indigenous communities as part of typical cuisine, in traditional sweets and cool drinks or as fodder for domestic animals; the dark to creamy-white seeds are consumed as a condiment for dishes or as snacks. Fruits are globular, oval-elliptical, oblong, or ovoid globe with a polar diameter of 20–50 cm, with an epicarp or rigid shell and creamy-white, lemon green to dark green coloration with white longitudinal stripes toward the apex; the pulp or mesocarp and endocarp are white with a granular-fibrous texture and a slightly sweet taste. Fruits have been used in traditional medicine to relieve gastrointestinal disorders, hemorrhoids, and fever and receive different names from the original peoples of Mexico (i.e., “tzilacayotli” from Nahuatl or “gueeto-xembe” from Zapoteco). A plant can produce up to 50 fruits with an average weight of 6 to 7 kg (4, 5) (Figure 1).

Leafy vegetables are sources of natural antioxidants such as phenolics, flavonoids, betalains, xanthophylls, violaxanthin, ascorbic acids, carotenoids with strong antioxidant potential, betacyanins, betaxanthins, chlorophyll a, chlorophyll b, and beta-carotenes that have high radical quenching ability. These are also sources of essential minerals, including K, Ca, Mg, P, and S, and microelements, including Fe, Cu, Mn, Zn, Na, Mo, and B; they also supply protein, dietary fiber, carbohydrates, and vitamins, for human nutrition (6–15).

Phytochemical analyses of *C. ficifolia* pulp and different biological tests *in vitro* and *in vivo* with extracts of this species suggest the wide potential that they may have in family community health improvement and in pharmacology. Fruits have shown a hypoglycemic preventive and corrective effect against type 2 diabetes mellitus (16). Moya-Hernández et al. (17) pointed out that the aqueous extracts of ripe fruits lower glucose in rats with induced diabetes and that the response is a product of the joint action of all the phenolic compounds rather than of individual compounds. In similar models, Alarcón-Aguilar et al. (18) also found a favorable response, but by oral rather than intraperitoneal administration. Banderas-Dorantes et al. (19) compared the hypoglycemic effect of the *C. ficifolia* extract in relation to the induced effect by glibenclamide in rat aortic rigs, where they showed that the effect is explained by the ATP mechanism-sensitive potassium channels. Diaz-Flores et al. (20) attributed the hypoglycemic effect to its antioxidant properties in the glutathione redox cycle. It was also proposed that the effect is due to the D-chiro-inositol content in the fruit (21). However, the study of the mechanisms of hypoglycemic action, mainly by using aqueous extracts, is still in progress.

Alshammari et al. (22) reported that *C. ficifolia* fruit extracts have antimutagenic or anticarcinogenic potential against MCF-7 breast cancer cells, and their observations show that they inhibit cell division and induce p53/caspase-mediated apoptosis. Roman-Ramos et al. (23) proposed that the content of D-chiro-inositol in the fruit generates high antioxidant and anti-inflammatory activity in rats with diabetes induced with streptozotocin. Xia and Wang (24) pointed out that treatment with aqueous extracts of *C. ficifolia* in rats with induced type 1 diabetes for 30 days generated a significant reduction in blood glucose, triglycerides, and low-density lipoprotein (LDL) and an increase in the level of high-density lipoprotein (HDL). Bayat et al. (25) found similar responses by combinations of *C. ficifolia* fruits and yogurt in induced type 2 diabetes. The mechanisms of action of extracts of *C. ficifolia* against diabetes based on their composition have not yet been clarified; therefore, studies with greater precision are needed, as well as a focus on other mechanisms such as hepatoprotective, anticancer, antimicrobial, and antiulcer activities (26), including changes in composition due to environmental, genotypic, or varietal interactions. In the present work, it was important to know if there are differences between physicochemical parameters and compound with antioxidant activity from “chilacayote” landraces, so the proposed objective was to evaluate the variation in physicochemical parameters, phenolic compound contents and antioxidant activity in the fruit mesocarp or pulp of a collection of landraces from Oaxaca, Mexico, the center of origin and diversification of *C. ficifolia*, for current and potential reassessment purposes of pre-Columbian Mesoamerican food origin.

## 2 Materials and methods

### 2.1 Origin of landraces evaluated

*Cucurbita ficifolia* fruits from different populations were collected in land parcels and backyards of farmers in the municipalities of San Martin Huamelúlpam (Huamelúlpam) and Santo Domingo Yanhuitlán (Yanhuitlán), Oaxaca, Mexico. Four to six fruits were collected in a mature state per population and farmer, harvested in the 2021 crop cycle and after transported to the laboratory. The municipalities have a sub-temperate to temperate climate with rainfall of 7,000–1,000 mm distributed from June to November, average annual temperatures of 14–18°C, and average altitudes of 2,200 m. Yanhuitlán is located at 17° 31′ 1″ LN and 97° 19′ 59″ LW, and Huamelúlpam is located at 17° 23′ 48″ LN and 97° 36′ 24″’ LW in southeastern Mexico within the region known as Mesoamerica and its communities identified with the Mixteco indigenous group. The *C. ficifolia* populations collected and evaluated were made up of 10 populations from Huamelúlpam (ID: 01–10 H) and 10 from Yanhuitlán (ID: 01–10 Y).

### 2.2 Sampling and extract preparations

Once the hard epicarp or shell was removed, the seedless fruit mesocarp was obtained to make five different extract types: Aqueous (5 mg with 10 mL of solvent), ethanolic (1 g in 30 mL of solvent), acetone (5 g in 10 mL of 70% solvent acidified to 1% with HCl), protein [1 g with 6 mg of PVPP (polyvinylpolypyrrolidone) in 25 mL of KCl buffer solution with pH 13] and juice (20 mL), small pieces of mesocarp were processed in an electric domestic extractor (Breville JE95XL, CA, USA). The aqueous, acetone and protein extracts, and juice, were homogenized at 50 rpm for 1 min (DAIHAN-Brand HG-15-A Gangwon, Korea) and after centrifuged (Hettich, Universal 32R, Tuttlingen, Germany) at 4,000 rpm for 20 min. The mixture was filtered through two layers of cheesecloth, and the filtrate was used for further analysis. The ethanolic extract was left to stand for 24 h and was later centrifuged and filtered for analysis.

### 2.3 Evaluation of chemical composition of pulp or mesocarp

*Soluble solid contents (SSC) and pH*: SSC content was analyzed in a digital refractometer (ATAGO, PR-32, Tokyo, Japan) and expressed as °Brix from 0.1 mL of the aqueous extract (27). The pH was measured in the aqueous extract using a potentiometer (Oakton 510, Vernon Hills, USA) according to the method described by the AOAC (28).

*Total sugars (TS)*: Total sugar content was evaluated by the phenol-sulfuric acid method described by Dubois et al. (29). A 1:20 dilution of the ethanolic extract was made from which 1 mL was taken and 100 μL of an 85% (p/v) phenol solution was added; then, it was homogenized with a vortex and 5 mL of H_2_SO_4_ conc. were added and homogenized again. It was incubated for 20 min in a water bath at 30°C, and the absorbance was read at 490 nm with a spectrophotometer (JENWAY 6305, Stafordshire, UK). Quantification was performed with reference to a standard calibration curve of anhydrous D-glucose (0.006–0.120 mg mL^–1^, *r*^2^ = 0.999). The results were expressed as mg of glucose per gram of sample on dry weight (mg G g^–1^ dw).

*Protein*: Protein content was estimated based on the method described by Bradford (30). A total of 500 μL of Bradford’s reagent was added to 500 μL of the extract, and the absorbance was read at 595 nm with a spectrophotometer. The concentration was calculated based on the bovine albumin standard curve (0.0005–0.018 mg mL^–1^, *r*^2^ = 0.995). The results were expressed as mg of bovine albumin (BA) per gram of sample on dry weight (mg BA g^–1^ dw).

*Total polyphenols*: Total polyphenol content was evaluated using the method described by Singleton and Rossi (31). First, 1 mL of distilled water and 200 μL of Folin–Ciocalteu were added and left to stand for 5–8 min. Then, 2 mL of 7% (w/v) Na_2_CO_3_ and 1.4 mL of distilled water were added to 400 μL of the acetone extract, vortexed and incubated for 1 h at room temperature, and the absorbance at 750 nm was measured using a spectrophotometer. Quantification was made with reference to the gallic acid standard curve (0.02–0.16 mg mL^–1^, *r*^2^ = 0.999), and the results were expressed in mg gallic acid equivalents per gram of sample on dry weight (mg GAE g^–1^ dw).

*Total flavonoids*: The determination of total flavonoid contents was made based on the method set up by Zhishen et al. (32). First, 75 μL of NaNO_2_ was added to 250 μL of the juice, incubated for 5 min and vortexed. Then, 150 μL of AlCl_3_ was added to the solution, and 1 min later, 500 μL NaOH was added to equal 3 mL with distilled water. The solution was stirred again, and finally, the absorbance at 510 nm was measured using a spectrophotometer. The flavonoid concentration was estimated based on the catechin standard curve (0.008–0.05 mg mL^–1^, *r*^2^ = 0.996), and the results were reported in mg equivalents of catechin per gram of sample on dry weight (mg CE g^–1^ dw).

*Antioxidant activity*: The antioxidant activity was evaluated by the DPPH (2,2-diphenyl-1-picrylhydrazyl) method described by Brand-Williams et al. (33) and by the FRAP method (ferric reducing antioxidant power) reported by Benzie and Strain (34). In the DPPH method, 2.9 mL of the DPPH solution was added to 100 μL of acetone extract in the dark. Subsequently, it was incubated at room temperature for 30 min, and the absorbance at 517 nm was recorded with a spectrophotometer. The reaction was estimated based on the Trolox standard curve (6-hydroxy-2,5,7,8-tetramethylchroman-2-carboxylic acid; 0.16–0.96 μmol mL^–1^, *r*^2^ = 0.997). In FRAP, the reaction of 100 μL of the acetone extract and 3 mL of FRAP reagent (300 mM C2H_3_NaO_2_, 10 mM TPTZ, FeCl_3_ 6H_2_O in a 10:1:1 ratio) was started. It was incubated for 30 min in a water bath at 37°C, and the absorbance at 593 nm was recorded with a spectrophotometer. Additionally, in this case, the reaction was estimated based on the Trolox standard curve (0.14–1.4 μmol mL^–1^; *r*^2^ = 0.999). In both cases, the results were reported as μmol equivalents of Trolox on dry weight (μmol TE g^–1^ dw).

### 2.4 Identification of phenolic compounds by ultra-performance liquid chromatography-mass spectrometry

Accelerated extraction was performed using solvents (ASE 350, Thermo Scientific, Sunnyvale, CA, USA) based on the method described by Juárez-Trujillo et al. (35) and Monribot et al. (36). A 300.6 mg sample of the lyophilized fruit mesocarp (10 H originating from Huamelúlpam) was mixed with 100 mg of diatomaceous soil. The cell was filled with methanol to a pressure of 1,500 psi and heated at 60°C for 5 min. The extract was concentrated in a rotary evaporator (Büchi RII, Switzerland). Subsequently, 100 mg of the dried methanolic extract was dissolved in methanol with 0.1% formic acid (MS grade, Sigma-Aldrich, St. Louis, MO, USA), filtered with 0.5 μm (PTFE Syringe Filter, Agilent, Palo Alto, CA, USA) and placed in 2 mL UPLS vials until analysis in triplicate.

Phenolic compound identification and quantification were performed with an ultrahigh resolution liquid chromatograph (UPLC, Agilent 1,290 infinity) coupled to a triple quadrupole mass spectrometer (MS-MS, Agilent 6460). Chromatographic analysis was performed with an Agilent Eclipse Plus C18 column (2.1 mm × 50 mm, 1.8 Microns) worked at 40°C. For the elution of compounds, water with 0.1% formic acid (A) and acetonitrile with 0.1% formic acid (B) were used as mobile phases. The gradient conditions were as follows: 0 min 1% B, from 0.1 to 40 min linear gradient 1–40% B, 40.1–42 min linear gradient 40–90% B, 42.1–44 min isocratic 90% B isocratic, 44.1–46 min linear gradient 90–1% B, 46.1–47 min 1% B isocratic (total run time 47 min). The flow rate was 0.3 mL min^–1^, and the injection volume was 2 μL.

The identification and quantification of phenolic compounds was determined according to the analytical conditions described by Juárez-Trujillo et al. (35), and the results were expressed as g per gram of sample in dry weight (μg g^−1^ dw).

### 2.5 Statistical analysis

Once the description database was integrated with the physicochemical parameters, phenolic compounds and antioxidant activity for each sample, analysis of variances was performed using a completely random linear model to evaluate the effect of municipalities of origin on the composition and differences between nested populations in municipalities. Subsequently, mean comparisons were made using the Tukey method (*p* < 0.05), all using the SAS statistical program (37).

## 3 Results

### 3.1 Analysis of variance

In the analysis of variance, significant differences (*p* < 0.05, 0.01) were found between the municipalities of origin of the samples in SSC, pH, total sugars, and flavonoid content. Among populations within the municipalities of origin, the differences were significant (*p* < 0.01) for all the variables evaluated. These results show that the variation in SSC, total sugars, and flavonoids among populations is greater than the variation between municipalities of origin of the populations, based on the variance estimated for each source of variation (Table 1).

**TABLE 1:** Significance of square means of the analysis of variance of physicochemical parameters, phenolic compounds, and antioxidant activity in the pulp of *C. ficifolia* fruits.

Parameters evaluated	Sources of variation				Coefficient variation (%)
	Origin municipalities (*M*)	Populations/M^1^	Replications	Error	
Soluble solid contents (°Brix)	10.08**	12.140**	3.570*	1.084	16.1
pH	0.462*	0.357**	0.038^ns^	0.074	5.0
Total sugars (mg G g^–1^)	0.027*	1.690**	1.120**	0.130	17.2
Protein (mg BA g^–1^)	0.074^ns^	0.188**	0.475**	0.049	13.5
Total polyphenols (mg GAE g^–1^)	0.002^ns^	0.580**	0.144*	0.046	13.2
Total flavonoids (mg CE g^–1^)	0.160*	0.753**	0.059^ns^	0.037	15.2
Ant. activity by FRAP (μmol TE g^–1^)	0.124^ns^	1.293**	0.177^ns^	0.132	13.7
Ant. activity by DPPH (μmol TE g^–1^)	0.020^ns^	0.838**	0.060^ns^	0.097	12.2

^1^Populations nested in origin municipalities; *Significant at *p* > 0.05; **significant at *p* < 0.05; ^ns^not significant (*p* > 0.05).

### 3.2 Chemical composition of mesocarp or pulp

Significant differences in SSC, TS, and pH were recorded between the municipality of origin, with a lower average concentration of soluble solids and sugars in Huamelúlpam and a slightly lower pH concentration in Yanhuitlán. In contrast, the amount of protein was similar between municipalities. The variation between populations in SSC, TS, and pH ranged from 4.30 (08 Y) to 8.97 (07 Y) °Brix, 0.40 (06 Y) to 0.70 (09 H) mg G g^–1^ and from 4.81 (07 Y) to 5.79 (01 H), respectively. In protein, the variation was from 5.02 (05 Y) to 15.31 (08 Y) mg BA g^–1^, with significant differences between populations within each municipality of origin (Table 2). Greater variation was also found between populations within the municipality of Yanhuitlán.

**TABLE 2:** Variation in soluble solid contents, pH, sugars, and protein content between *C. ficifolia* populations from two municipalities of Oaxaca, Mexico.

ID populations/ origin municipalities	Soluble solid contents (°Brix)	pH	Total sugars (mg G g^–1^)	Protein (mg BA g^–1^)
01H	6.93 bc^1^	**5.79 a**	0.50 e–h	10.68 a–d
02H	6.17 bcd	5.26 bcd	0.43 gh	7.69 bcd
03H	5.67 b–e	5.61 ab	0.44 f–h	11.42 abc
04H	5.40 cde	5.35 abc	0.53 c–g	5.85 bcd
05H	6.00 b–e	5.28 bc	0.48 e–h	7.82 bcd
06H	5.87 b–e	5.32 bc	0.51 e–h	7.54 bcd
07H	4.90 de	5.30 bc	0.64 a–d	6.45 bcd
08H	7.27 ab	5.39 abc	0.50 e–h	7.78 bcd
09H	6.70 bc	5.59 abc	**0.70 a**	6.93 bcd
10H	7.30 ab	5.48 abc	0.50 e–h	6.49 bcd
*Huamelulpam (average)*	6.22 B^1^	5.44 A	0.52 B	7.90 A
01Y	7.40 ab	5.28 bc	0.59 a–e	6.70 bcd
02Y	5.47 cde	5.41 abc	0.43 gh	9.96 a–d
03Y	**8.77 a**	5.39 abc	0.52 d–h	5.59 cd
04Y	6.80 bc	5.53 abc	0.56 b–f	6.53 bcd
05Y	6.00 b–e	5.13 cd	0.55 b–g	5.02 d
06Y	5.87 b–e	5.36 abc	0.40 h	10.28 a–d
07Y	**8.97 a**	4.81 d	0.65 a–c	6.74 bcd
08Y	4.30 e	5.56 abc	0.52 d–h	**15.31 a**
09Y	6.77 bc	5.43 abc	0.58 a–e	8.81 bcd
10Y	6.60 bcd	5.44 abc	0.67 ab	11.93 ab
*Yanhuitlan (average)*	6.69 A	5.33 B	0.55 A	8.69 A

^1^In column, means of populations or origin municipality with same letter (lower or upper case) are not significantly different (Tukey’s test, *p* < 0.05).

The bold numbers indicate the highest value, and the underlined numbers indicate the lowest value for each parameter.

In total polyphenol contents and antioxidant activity, no significant differences were found (*p* > 0.05) between municipalities of origin of the evaluated populations; in flavonoids, the average of Yanhuitlán was higher than that of Huamelúlpam. Between populations, the variation in the total polyphenol and flavonoid concentrations ranged from 1.94 (05 Y) to 4.89 (06 Y) mg GAE g^–1^ and from 0.86 (03 Y) to 1.87 (08 Y) mg CE g^–1^, respectively. The antioxidant activity varied from 4.36 (08 H) to 10.63 (06 H) and from 4.57 (03 Y) to 12.59 (08 Y) μmol TE g^–1^ evaluated by DPPH and FRAP methods, respectively (Table 3). The variation between populations within the municipalities of Huamelúlpam and Yanhuitlán in total polyphenols was equivalent, 1.97–4.83 and 1.94–4.89 mg GAE g^–1^, respectively, but not in total flavonoid concentrations with values of 1.05–1.54 and 0.86–1.87 mg CE g^–1^, respectively, and shows that the landrace and area of origin influence the fruit mesocarp composition of *C. ficifolia*.

**TABLE 3:** Variation in total polyphenol and flavonoid contents and antioxidant activity between *C. ficifolia* populations from two municipalities of Oaxaca, Mexico.

ID populations/ origin municipalities	Total polyphenols (mg GAE g^–1^)	Total flavonoids (mg CE g^–1^)	Antioxidant activity (μ mol TE g^–1^)	
			DPPH	FRAP
01H	2.79 c^1^	1.29 c–f	6.51 bcd	7.26 bcd
02H	2.41 c	1.09 efg	5.86 bcd	6.81 bcd
03H	1.97 c	1.48 bcd	6.40 bcd	6.08 cd
04H	2.12 c	1.51 bc	8.16 abc	9.38 abc
05H	2.68 c	1.05 efg	6.09 bcd	4.91 d
06H	4.62 ab	1.54 abc	**10.63 a**	**11.78 a**
07H	2.06 c	1.13 efg	8.34 ab	7.53 bcd
08H	2.25 c	1.06 efg	4.36 d	4.79 d
09H	2.29 c	1.09 efg	6.02 bcd	6.34 bcd
10H	**4.83 a**	1.17 d–g	5.58 bcd	7.20 bcd
*Huamelulpam (average)*	2.80 A^1^	1.24 B	6.81 A	7.21 A
01Y	2.47 c	1.62 abc	5.97 bcd	6.97 bcd
02Y	2.14 c	0.99 fg	6.36 bcd	7.08 bcd
03Y	2.47 c	0.86 g	5.35 cd	4.57 d
04Y	2.65 c	1.50 bcd	5.78 bcd	6.92 bcd
05Y	1.94 c	1.37 cde	5.75 bcd	5.69 d
06Y	**4.89 a**	0.94 g	7.82 abc	9.65 ab
07Y	3.30 bc	1.13 efg	5.39 cd	7.15 bcd
08Y	2.66 c	**1.87 a**	**10.52 a**	**12.59 a**
09Y	2.43 c	1.71 ab	7.35 bc	7.92 bcd
10Y	2.54 c	1.03 fg	5.51 bcd	6.01 cd
*Yanhuitlan (average)*	2.75 A	1.30 A	6.58 A	7.45 A

^1^In column, means of populations or origin municipality with same letter (lower or upper case) are not significantly different (Tukey’s test, *p* < 0.05).

The bold numbers indicate the highest value, and the underlined numbers indicate the lowest value for each parameter.

**FIGURE 2:**
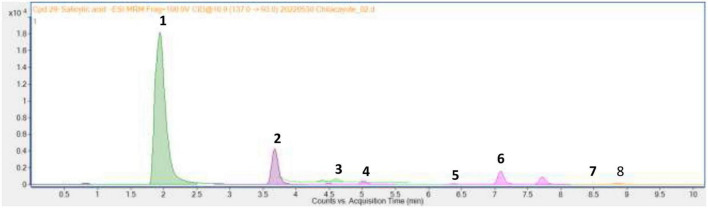
Chromatogram of methanol extract of *C. ficifolia* pulp where: 1, L-phenylalanine; 2, 4-hydroxybenzoic acid; 3, 4-hydroxyphenylacetic acid; 4, vanillic acid; 5, vanillin; 6, 4-coumaric acid; 7, ferulic acid; 8, ferulic acid.

Through UPLC-MS analysis, the following compounds were identified: L-phenylalanine, 4-hydroxybenzoic acid, 4-hydroxyphenylacetic acid, vanillic acid, vanillin, 4-coumaric acid, ferulic acid, and salicylic acid, with concentrations of 230.68 ± 14.9, 11.70 ± 0.1, 1.70 ± 0.1, 0.57 ± 0.03, 0.12 ± 0.01, 2.04 ± 0.11, 0.05, and 0.10 ± 0.01 μg g^–1^, respectively (Figure 2). These are an essential amino acid (L-phenylalanine), a phenolic aldehyde (vanillin), and six phenolic acids that contribute to the antioxidant capacity and functional properties of the fruit mesocarp of *C. ficifolia.*

## 4 Discussion

### 4.1 Chemical composition of mesocarp or pulp

In Mexico, Central and South America, Europe and Asia, the immature and ripe fresh fruits of *C. ficifolia* are consumed (4, 5), but it continues to be underutilized, unknown or consumed less often than the fruits of *C. pepo*, *C. moschata*, and *C. maxima*. Moya-Hernández et al. (17) showed that *C. ficifolia* fruits undergo different physiological processes from a few grams (primordia) to more than 7.2 kg in weight (>45 g after anthesis), and consequently, their external and internal composition changes, including the seed composition (38). That is, by changing the fruit composition, they can change their current and potential nutritional-nutraceutical value. In the present study, all the fruits evaluated were ripe with a rigid external epicarp and varied in weight from 7 to 9 kg. The average values of SSC in the fruits collected in Huamelúlpam and Yanhuitlán ranged from 4.30 to 8.97 °Brix, similar to values estimated by Moya-Hernández et al. (17), where these authors recorded a variation of 5.0–6.0 °Brix in the fruit’s development stage from 10 to 55 days after anthesis and slightly different from the interval of the present work of 4.30–8.97 °Brix. The estimation of SSC shows the amount of sugars and organic acids in the fruit pulp. In this work, the sugar concentration was similar to the average estimated by Jacobo-Valenzuela et al. (39) in *C. moschata* fruits (6.42 ± 2.26 °Brix) but slightly higher than the values reported by Martínez-Valdivieso et al. (40) in different landraces of *C. pepo* (3.4–4.7 °Brix) and lower than the values determined by Biesiada et al. (41) in *C. maxima* (9.4–10.9 °Brix) under increased application of organic matter (manure). The results show that the sugars contained in the *C. ficifolia* fruits provide a certain palatability similar to that of other cucurbits used fresh (vegetable) or processed during the preparation of traditional dishes.

The mesocarp of *C. ficifolia* fruits was acidic since among populations, it varied from 4.81 to 5.79, slightly lower than the range reported by Martínez-Valdivieso et al. (40) for *C. pepo* landraces (6.5–6.9) and for *C. moschata* (6.77 ± 0.7) registered by Jacobo-Valenzuela et al. (39), but similar to the records of Moya-Hernández et al. (17) in *C. ficifolia* (5.4–6.1). In this work, each population had its own patterns, and the pH showed a proportional relationship to the total sugar amount (0.2 ≤ *r* ≤ 0.7), for example, in the cases of 1 H, 03 H, 04 Y and 06 Y. In other cases, the relationship was similar in acidity or pH, and sugar content influenced the taste perception for *C. ficifolia* fruit consumers and could influence food preferences. The sugar content varied from 0.40 to 0.7 mg G g^–1^, slightly lower than that reported by Bressani (42), 0.702–0.777 mg G g^–1^.

The amount of protein in fruit pulp varied from 5.02 to 15.31 mg AB g^–1^, where four native populations from Yanhuitlán had contents higher than 9.95 mg AB g^–1^, but only two populations from Huamelúlpam exceeded that value. This is relevant from the nutritional point of view because it is possible to select populations among farmers with high protein values. The results are lower than the values reported in *C. moschata* (165.3 mg of AB g^–1^) and *C. maxima* (182 mg of AB g^–1^) using the Kjeldahl method (43, 44). Men et al. (45) pointed out that the protein content in *C. moschata* stands for 0.8–19.6%, but for the same species, Jacobo-Valenzuela et al. (39) showed that they represent 1.4%. Based on the proportionality showed for *C. moschata* and respecting the differences between species, it is possible to infer that *C. ficifolia* can supplement the diet for protein, among other contributions.

In different plant-based foods, the bioactive compounds and antioxidant activity are responsible of the functional activity once they are consumed more regularly or are included in a greater proportion of daily diets. Phenolics include coumarins; phenolic acids, such as hydroxybenzoic acids and hydroxycinnamic acids; flavonoids, such as flavonols, flavones, flavanols, flavanones, isoflavones, anthocyanins, and chalcones; and nonflavonoids, such as tannins, lignans, and stilbenes (46–51).

In *C. ficifolia* pulp, a variation in total polyphenol content was determined from 1.94 to 4.89 mg GAE g^–1^ dw. These values are consistent with the estimates of Moya-Hernández et al. (7), who shown that the concentration of gallic acid in *C. ficifolia* decreases from 16.97 to 0.24 mg g^–1^ from 15 to 45 days after anthesis (settling to fruit ripening). The values reported here are significantly lower than those reported by Oyeleke et al. (52) for *C. maxima* and *C. mixta* (30–45 mg GAE g^–1^ dw), while those determined by Hussain et al. (53) in *C. maxima* (1.34 ± 0.2 mg GAE g^–1^ dw) are slightly higher, as are the records of Priori et al. (54) in *C. moschata* (0.26–0.79 mg GAE g^–1^ fw). Similarities and differences in *C. ficifolia* polyphenols with respect to other cucurbits depend on distinct factors, such as the variety, stage of fruit development, plant growth conditions, laboratory methodologies, and their interactions. However, the food contribution of *C. ficifolia* fruit for consumers and its potential effects on health are relevant.

The pulp flavonoid content varied from 0.86 to 1.87 mg CE g^–1^, lower values than those found by Oyeleke et al. (52) in *C. mixta* and *C. maxima* (5–9 mg QE g^–1^) and by Mokhtar et al. (55) in *C. moschata* (28.66 mg QE g^–1^). Although these values are reported in terms of quercetin equivalents (QE), they are similar to those reported in catechin equivalents (CE), such as the records of Jacobo-Valenzuela et al. (39) in *C. moschata* (1.38 ± 0.54 mg CE g^–1^) and by Hussain et al. (53) in *C. maxima* (0.77 mg CE g^–1^). In these and other cases, the laboratory protocols, reference standards and extraction solutions used influence the flavonoid estimations. For example, Enneb et al. (44) determined significant differences in the estimation of *C. moschata* pulp flavonoids comparing the use of methanol (close to 30 mg QE g^–1^) vs. hexane, chloroform, and acetate (5–12 mg QE g^–1^) with a quercetin standard base. Despite the differences in estimated values, *C. ficifolia* pulp can contribute flavonoids to the diet, which have an antioxidant effect on the health of consumers and have benefits against coronary heart disease prevention and the antimutagenicity of certain cancer cells and antiviral processes are still under study (56).

The identification of six phenolic compounds, a vanillin and an amino acid by UPLC-MS in *C. ficifolia* pulp helps to describe the composition and its potential effects for consumers. L-phenylalanine, 4-hydroxybenzoic acid, 4-hydroxyphenylacetic acid, and 4-coumaric acid were the most relevant compounds in relation to the concentration in the evaluated population (230.7–1.7 μg g^–1^). These registered compounds differ from most compounds found by Enneb et al. (44) by LC-EST-MS in *C. moschata*: quinic acid, syringic acid, p-coumaric acid and trans-cinnamic acid. In contrast, this agrees with Mokhtar et al. (55) who found in vanillin and ferulic acid from a total of 33 phenolic compounds determined in *C. moschata* by HPLC-DAD-ESI-MS, and in 4-hydroxybenzoic acid and ferulic acid determined by HPLC-DAD in *C. moschata* and *C. pepo* by Kulczynski and Gramza-Michalowska (57). In fruit pulp of *C. ficifolia*, Moya-Hernández et al. (17) determined the presence of gallic acid, chlorogenic acid, catechin, epicatechin, syringic acid, and myricetin by HPLC-DAD, and Fortis-Barrera et al. (21) found D-chiro-inositol in *C. ficifolia pulp* extracts. All these compounds confer anti-inflammatory and antioxidant activity ability in oxidative stress conditions. This makes the complex of phenolic compounds present in the pulp of *C. ficifolia* confer functional and beneficial properties against risk factors for diabetes and other chronic degenerative diseases (55, 58, 59).

The antioxidant activity varied from 4.36 to 10.63 and from 4.57 to 12.59 μmol TE g^–1^ dw, based on DPPH and FRAP methods, respectively. These are higher values than those recorded by Mokhtar et al. (39) in *C. moschata* (0.048–0.065 μmol TE g^–1^ dw), by Kulczynski et al. (59) for *C. moschata* and *C. pepo* (0.48–1.50 μmol TE g^–1^ dw) and by Priori et al. (54) in *C. moschata* (0.71–3.58 μmol TE g^–1^ fw), all evaluated by the DPPH method. By the FRAP method, Kulczynski et al. (59) recorded variations from 1.89 to 5.16 μmol TE g^–1^ dw in *C. moschata* and *C. pepo*, and Nawirska-Olszanska et al. (60) determined values from 2.08 to 8.07 μmol TE g^–1^ dw in *C. moschata* and in *C. ficifolia* that recorded 2.10–2.27 μmol TE g^–1^ dw, values that coincide with part of this work. This shows that *C. ficifolia* pulp could be an important source of compounds with antioxidant activity, which could have an effect on factors that induce oxidative stress.

Physicochemical parameter analysis and the evaluation of phenolic compounds and antioxidant activity in 20 populations of *C. ficifolia* showed that the geographical origin and the populations preserved by small farmers present differences and high variability in pulp composition and can influence the potential benefits of its frequent consumption. The identification of eight compounds by UPLC-MS shows nutritional-nutraceutical potential. For example, L-phenylalanine and 4-hydroxybenzoic acid, are the main compounds identified and quantified in this work. Fortis-Barrera et al. (21) point out that the antioxidant and anti-inflammatory effect obtained *in vitro* with the application of *C. ficifolia* extracts is explained, in part, by its content of D-chiro-inositol, but they do not rule out that it is the product of other compounds present in the pulp. This same processing of *C. ficifolia* extract was used by Roman-Ramos et al. (23) in biological models in rats with induced diabetes and oral administration of the extract, where they concluded that it helps control diabetes mellitus and has hypoglycemic activities. Bayat et al. (25) pointed out that *C. ficifolia* juice combined with yogurt favored blood sugar control, LDL cholesterol reduction, and anti-inflammatory activity in the control of type 2 diabetes.

Alashammari et al. (22) used whole fruit flour and chloroform extraction, with later elimination, and found D-glucopyranosylamine, n-hexadecanoic and 1–4-cyclooctadiene as major components, then evaluated their effect on the growth of breast cancer cells and found that it induces MCF-7-cell death because it inhibits cell division and is proposed as a preventive agent. In these and other cases, aqueous or methanolic extracts are evaluated with more regularity using the seedless pulp or juice, but without isolation of specific compounds, and the results support the hypoglycemic effect and factors related to diabetes (19, 20, 24). These advances also show that it is necessary to continue expanding the knowledge of the *C. ficifolia* fruit composition and to evaluate its potential health effects.

## 5 Conclusion

We found wide variation in physicochemical parameters (SSC, pH, total sugars, and protein), total polyphenols and flavonoids, and antioxidant activity (evaluated by DPPH and FRAP methods) in fruits of 20 *C. ficifolia* native populations collected through two municipalities from Oaxaca, México (Huamelúlpam and Yanhuitlán). In addition, eight compounds were identified by UPLC-MS: L-phenylalanine (amino acid), vanillin (phenolic aldehyde), and six phenolic acids (4-hydroxybenzoic acid, 4-hydroxyphenylacetic acid, vanillic acid, 4-coumaric acid, ferulic acid, and salicylic acid), all with potential health effects. Specifically, L-phenylalanine and 4-hydroxybenzoic acid were the compounds with major concentrations, based on UPLCS-MS evaluation, and they have nutritional potential. Consequently, these results encourage us to recommend *C. ficifolia* fruit pulp consumption due to their potential effects on health as well as complement earlier studies about their composition and their effects on diabetes control.

## Data availability statement

The original contributions presented in this study are included in the article/supplementary material, further inquiries can be directed to the corresponding author.

## Author contributions

JA-J, EA-B, and JC-S: conceptualization and methodology. GM-Q, JA-J, and JC-S: investigation and writing. All authors read and agreed to the published version of the manuscript.
